# Evaluation of Total Eosinophil Counts, Serum Allergen-Specific IgE and Related Cytokines in Dogs with Atopic Dermatitis

**DOI:** 10.3390/ani15213219

**Published:** 2025-11-05

**Authors:** Min-Joo Chae, Min-Hee Kang, Hee-Myung Park

**Affiliations:** 1 Cheonan Animal Medical Center, Cheonan 31155, Republic of Korea; erom5@daum.net; 2 Department of Veterinary Internal Medicine, College of Veterinary Medicine, Konkuk University, Seoul 05029, Republic of Korea; 3 Department of Bio-Animal Health, Jangan University, Hwaseong 18331, Republic of Korea; mhkang@jangan.ac.kr

**Keywords:** canine allergy, immunoglobulin E, cytokine profiling, hypersensitivity reactions, biomarker assessment

## Abstract

**Simple Summary:**

Canine atopic dermatitis (AD) is a common allergic skin disease that often causes itching and discomfort in dogs, leading to reduced quality of life. To better understand the usefulness of blood-based tests in dogs with AD, we examined eosinophil counts, serum allergen-specific immunoglobulin E (IgE), and several cytokines related to allergy and immune regulation. Ninety-three dogs were enrolled, including 65 diagnosed with AD and 28 healthy controls. We found that eosinophil counts were not significantly different between affected and healthy dogs, suggesting limited diagnostic value. However, allergen-specific IgE testing showed higher levels and sensitization rates to several common environmental and food allergens, particularly house dust and storage mites, pollens, and certain dietary ingredients. Cytokine levels showed some trends but were not significantly different. Our results indicate that allergen-specific IgE testing can provide meaningful information for diagnosis and management of AD in dogs.

**Abstract:**

Canine atopic dermatitis (AD) is a chronic allergic skin disease in which various immunological markers have been investigated. While peripheral eosinophil counts, serum allergen-specific immunoglobulin E (IgE), and cytokines have each been evaluated in allergic disorders, their simultaneous assessment in dogs with AD has rarely been reported in Korea. This study aimed to evaluate the diagnostic and clinical utility of these parameters in affected dogs. A total of 93 dogs were included between August 2019 and February 2020, comprising 65 dogs diagnosed with AD and 28 healthy controls. Clinical information, peripheral blood eosinophil counts and ratios, serum allergen-specific IgE using a multiple allergen panel (60 allergens), and cytokines related to T helper 2 (Th2) and T regulatory (Treg) cells (IL-4, IL-13, IL-31, TGF-β1) were analyzed. The mean age of AD dogs was 6.34 ± 3.99 years, with a predominance of small breeds and males. Eosinophil counts and ratios showed no significant difference between groups. In contrast, allergen-specific IgE levels were significantly elevated for several allergens, including *Dermatophagoides pteronyssinus*, *Acarus siro*, *Tyrophagus putrescentiae*, alder/birch, hazel, oak, cladosporium, and selected dietary antigens (pea, soybean, pumpkin, apple) (*p* < 0.05). Sensitization rates were also higher for *Acarus siro, Tyrophagus putrescentiae*, oak, and sheep sorrel (*p* < 0.05). Th2-related cytokines tended to increase and TGF-β1 tended to decrease in AD dogs, though without statistical significance. These findings indicate that peripheral eosinophil counts have limited diagnostic value, whereas allergen-specific IgE testing provides clinically useful information for the diagnosis and management of canine AD. Further research stratifying disease stages and assessing local tissue cytokine expression is warranted.

## 1. Introduction

Atopic dermatitis (AD) is a chronic, relapsing, and pruritic skin disease that markedly impairs quality of life in affected dogs and their owners [1]. Its immunopathological mechanisms parallel those in humans, making it an important comparative model for allergic skin disease [2]. In people, allergic disorders such as asthma, allergic rhinitis, and AD affect up to 30% of the population, particularly in children [3]. In dogs, AD accounts for an estimated 10–15% of cases, representing one of the most frequent dermatologic problems in small-animal practice [2,4].

AD is a multifactorial disorder involving genetic susceptibility, immune dysregulation, epidermal barrier impairment, and environmental triggers [2,5]. Because no single test is definitive, structured clinical assessment remains central to diagnosis. International guidelines, such as those of the International Committee for Allergic Diseases in Animals (ICADA), recommend standardized criteria including Favrot’s criteria, alongside the exclusion of other pruritic dermatoses [4,6]. Once clinical diagnosis is established, allergen identification by intradermal testing (IDST) or serum allergen-specific immunoglobulin E (IgE) assays is recommended to guide allergen-specific immunotherapy [7].

Peripheral eosinophilia, while often present in human allergic diseases, has shown inconsistent association with canine AD and therefore provides limited diagnostic value [8]. Likewise, total serum IgE concentrations vary widely among individuals, restricting its usefulness as a standalone diagnostic marker [9]. By contrast, allergen-specific IgE testing has become a more reliable approach for identifying sensitization patterns in dogs, with comparable clinical utility to IDST in guiding allergen-specific immunotherapy [10].

**Figure 1:**
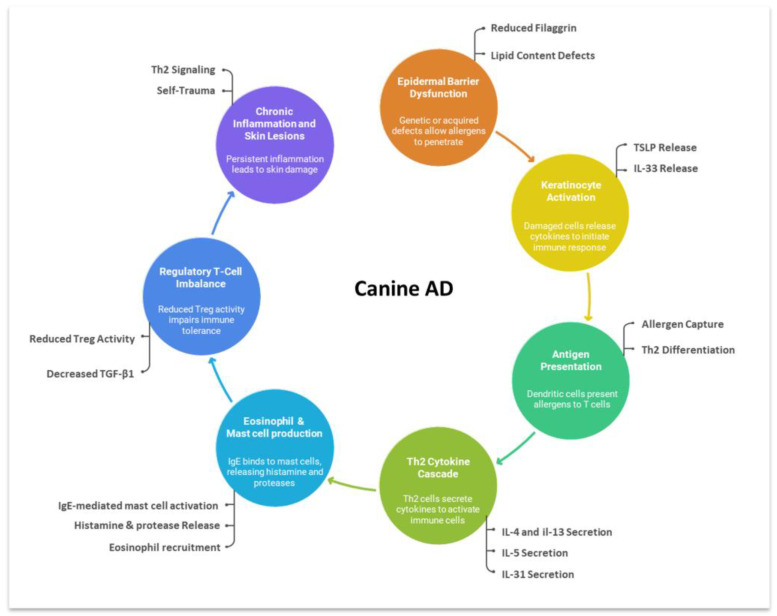
Schematic overview of the immunopathogenesis of canine AD. Epidermal barrier defects allow allergen penetration and keratinocyte activation. Released cytokines such as thymic stromal lymphopoietin (TSLP) and IL-33 stimulate dendritic cells to drive Th2 differentiation, followed by secretion of IL-4, IL-5, IL-13, and IL-31. These cytokines promote IgE-mediated mast-cell activation, eosinophil recruitment, and pruritic inflammation. Reduced Treg activity and decreased TGF-β1 impair immune tolerance, sustaining chronic inflammation and recurrent skin lesions.

Cytokines produced by T helper (Th) and regulatory T (Treg) cells are central to the immunopathogenesis of AD. Th2 cytokines such as interleukin (IL)-4, IL-13, and IL-31 promote inflammation and pruritus, whereas regulatory cytokines including transforming growth factor-β1 (TGF-β1) help preserve immune homeostasis [11,12]. The development of targeted biologics against these pathways highlights their translational importance in both human and veterinary medicine [13,14,15]. A schematic overview of the immunopathogenesis of canine AD, including barrier dysfunction, allergen sensitization, and Th2/Treg cytokine imbalance, is presented in Figure 1 to facilitate understanding of the mechanisms discussed in this study.

Despite the clinical importance of these markers, few studies have simultaneously assessed eosinophil counts, allergen-specific IgE, and cytokine profiles in canine AD. The present study aimed to address this gap by evaluating these parameters in dogs with AD compared with healthy controls, thereby assessing their diagnostic value and potential utility in guiding management strategies.

## 2. Materials and Methods

### 2.1. Study Population

Client-owned dogs presented to Cheonan Animal Medical Center between August 2019 and February 2020 were prospectively enrolled. The AD group included dogs that fulfilled the clinical diagnostic criteria for AD, underwent blood sampling prior to initiation of any treatment, and had written owner consent for the use of residual samples. Diagnosis was established based on history, pruritus, lesion distribution, chronicity, and exclusion of other pruritic dermatoses, with at least five of Favrot’s criteria required [6]. Dogs with food-induced AD were included if they fulfilled Favrot’s clinical criteria for AD. Blood samples were collected prior to initiation of any treatment, and written owner consent was obtained for the use of residual samples.

The control group consisted of clinically healthy dogs with no history of pruritus, allergic disease, or chronic dermatologic conditions, recruited during routine health checks or elective neutering. Written owner consent was obtained. Exclusion criteria included prior administration of antihistamines, corticosteroids, cyclosporine, oclacitinib, or lokivetmab within one month before enrollment, as well as the presence of systemic illnesses other than AD.

### 2.2. Sample Collection and Storage

Approximately 3–5 mL of blood was collected from the jugular vein using aseptic technique under manual restraint without sedation. Samples were divided into EDTA tubes (for hematology) and serum-separating tubes (SST). Complete blood counts were performed within 30 min of collection to minimize storage-related artifacts. Serum was separated by centrifugation at 3000 rpm for 5 min, aliquoted into plain tubes, and stored at −20 °C until batch analysis. The storage period did not exceed 3 months.

### 2.3. Eosinophil Counts

Absolute eosinophil counts and percentages were measured using an automated hematology analyzer (ADVIA 2120i, Siemens, Erlangen, Germany), which has been validated for canine hematology. Reference intervals (0–0.6 × 10^9^/L for absolute counts, 0–7% for percentages) were based on manufacturer-provided data and are consistent with published canine hematology ranges. Both absolute counts and percentages were analyzed statistically, although absolute counts were considered the primary parameter. Manual blood smears were reviewed in a subset of samples to verify leukocyte differentials.

### 2.4. Allergen-Specific IgE Assay

Serum allergen-specific IgE was quantified using a commercial multiple allergen simultaneous test–immunoblot (MAST-immunoblot; ANITIA Canine IgE, Proteome-Tech, Seoul, Republic of Korea) according to the manufacturer’s protocol. All assays were performed in duplicate, and mean values were used for analysis. To assess reproducibility, 15 serum samples were randomly selected and retested, and agreement was evaluated using the intraclass correlation coefficient (ICC); values ≥ 0.75 were considered indicative of good reliability.

The allergen panel comprised 60 antigens: 31 environmental (house dust mites, storage mites, pollens, fungi, insects, others) and 29 dietary (meat, grains/legumes, fruits/vegetables, yeast, dairy, seafood, nuts). A full list of allergens is provided in Supplementary Table S1. Results were scored on a 0–6 scale according to the alternate scoring method (ASM) [16,17]. Values ≥ Class 2 (≥0.35 AU/mL) were considered positive, following the manufacturer’s guideline and consistent with previously published canine studies using the same cutoff [17]. The ASM classification system and interpretation criteria are summarized in Table 1.

### 2.5. Cytokine Assays

Serum concentrations of IL-4, IL-13, IL-31, and TGF-β1 were determined using commercial enzyme-linked immunosorbent assay (ELISA) kits (IL-4, IL-13, IL-31: ABclonal, Woburn, MA, USA; TGF-β1: R&D Systems, Minneapolis, MN, USA) according to the manufacturers’ protocols. Each sample was analyzed in duplicate, and both intra- and inter-assay coefficients of variation were <10%. Serum aliquots were initially stored at −20 °C for ≤1 week immediately after processing and subsequently transferred to −80 °C for long-term storage (up to 6 months) before analysis. All assays were performed after a single freeze–thaw cycle to minimize cytokine degradation. The ELISA kits used were validated for canine use or had manufacturer-confirmed cross-reactivity, and their analytical performance (limit of detection and coefficient of variation) was within manufacturer specifications.

### 2.6. Statistical Analysis

Data are presented as median [interquartile range (IQR)] unless otherwise specified. Normality of continuous variables was assessed using the Kolmogorov–Smirnov test together with evaluation of skewness and kurtosis. As most allergen concentrations exhibited non-normal distributions with right-skewed patterns, non-parametric methods were primarily applied. Between-group comparisons of eosinophil counts, allergen-specific IgE concentrations, and cytokine levels were performed using the independent *t*-test or the Mann–Whitney U test, whereas normally distributed variables, if any, were analyzed using the independent t-test. For multiple allergen comparisons of IgE concentrations, Bonferroni correction was applied to reduce the risk of type I error. The prevalence of positive allergen responses (≥Class 2) was compared using chi-square or Fisher’s exact test, and the strength of association was expressed as odds ratios (OR) with 95% confidence intervals (CI) using binary logistic regression. Additionally, multivariate logistic regression analysis was performed to identify independent associations between allergen positivity and atopic dermatitis, adjusting for age, sex, breed, and neutering status. Reliability of the allergen-specific IgE assay was evaluated using the ICC, with values ≥ 0.75 considered indicative of good agreement. Statistical significance was set at *p* < 0.05. Analyses were performed using SPSS Statistics version 20.0 (IBM Corp., Armonk, NY, USA).

## 3. Results

### 3.1. Characteristics of the Study Population

A total of 111 dogs were evaluated in this study. Among them, 65 dogs fulfilled the clinical and inclusion criteria for atopic dermatitis after excluding those with comorbidities or recent medication use, and 28 clinically healthy dogs were enrolled as controls. Demographics of the AD and control groups are summarized in Table 2. The mean age was 6.34 ± 3.99 years in the AD group and 3.59 ± 4.12 years in the control group, with a younger distribution in controls (43% < 1 year) compared with AD dogs (52% between 1 and 6 years). Breed distribution was dominated by small companion breeds, especially Maltese (20 in AD; 6 in controls), with additional representation of Shih Tzu, Poodle, and Yorkshire Terrier in the AD group, and Poodle, Pomeranian, and Bichon Frise in controls.

### 3.2. Peripheral Eosinophil Counts

Peripheral blood eosinophil values were assessed in both groups. In the AD group, the absolute eosinophil count was 0.32 ± 0.29 × 10^9^/L (median 0.225, range 0–1.4), compared with 0.49 ± 0.87 × 10^9^/L (median 0.33, range 0.03–4.7) in controls. Both distributions were largely within the reference interval (0–0.6 × 10^9^/L), and no significant difference was observed (*p* = 0.945).

Relative eosinophil percentages were also analyzed, yielding 3.39 ± 2.82% (median 3.0, range 0.2–11.5) in the AD group and 3.72 ± 2.91% (median 3.4, range 0.2–12.9) in controls. These values were mostly within the reference range (0–7%), and no significant difference was detected (*p* = 0.621). Both absolute counts and percentages were statistically compared, although absolute counts were considered the primary parameter.

### 3.3. Serum Allergen-Specific IgE

The reliability of the commercial MAST-immunoblot was confirmed by duplicate testing of 15 randomly selected samples, yielding an ICC of 0.948 (95% CI: 0.940–0.954, *p* < 0.001), indicating excellent reproducibility. Comparisons of allergen-specific IgE concentrations between groups revealed significantly higher levels in the AD group for several environmental allergens, including *Dermatophagoides pteronyssinus* (d1), *Acarus siro* (d70), and *Tyrophagus putrescentiae* (d72). Among pollens, alder/birch (t2/t3), hazel (t4), maple leaf sycamore (t11), willow/cottonwood (t12/t14), oak (t7), plantain (w9), and sheep’s sorrel (w81) all showed significant elevations in AD dogs (all *p* < 0.05). *Cladosporium herbarum* (m3) and cat dander (e1/e2) also differed significantly in serum IgE concentrations, while no group differences were found for insect allergens. For dietary allergens, significant increases were detected in pea (f12) and soybean (f14) within legumes, and pumpkin (f225) and apple (f49) within fruits/vegetables, whereas meat, dairy, seafood, yeast, and nut allergens showed no significant differences (Table 3).

Positivity analysis, defined as ≥Class 2 on the alternate scoring method, revealed significant differences for four allergens. Among mites, *Acarus siro* (d70) was positive in 21.5% of AD dogs compared with 3.6% of controls (*p* = 0.033), while *Tyrophagus putrescentiae* (d72) showed positivity in 23.1% of AD dogs versus 3.6% of controls (*p* = 0.033). Within pollens, oak (t7) was positive in 16.9% of AD dogs but absent in controls (0%; *p* = 0.030), and sheep’s sorrel (w81) was positive in 27.7% of AD dogs compared with 7.1% of controls (*p* = 0.029). No other allergens, including dietary antigens, demonstrated significant group differences in positivity. Several allergens—including duck meat (f581), turkey meat (f284), yeast (f45), and mackerel (f206)—were consistently negative in both groups. These detailed positivity rates and corresponding OR and 95% CI for each allergen are provided in Supplementary Table S2.

Odds ratio and relative risk analyses (Table 4) confirmed that storage mite sensitization was markedly higher in AD dogs (ORs 7.4 and 8.1, respectively), while relative risks were moderate (approximately 1.4), indicating that although the odds were greatly increased, the actual probability difference between groups remained moderate.

To further determine whether these associations remained significant after controlling for confounding factors, a multivariate logistic regression analysis was performed including age, sex, breed, and neutering status as covariates. The results showed that storage mite sensitization, particularly *Acarus siro* (d70), remained independently associated with AD after adjustment, while *Tyrophagus putrescentiae* (d72) demonstrated a marginal trend. In contrast, pollen allergens (Ryegrass, Cultivated rye) were not significantly associated with AD after adjustment (Table 5).

**Figure 2:**
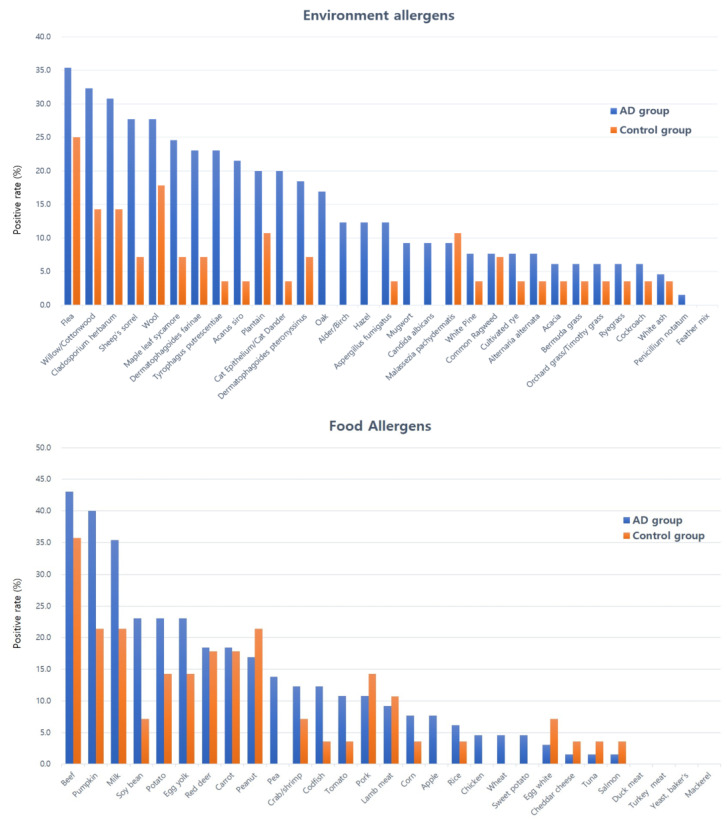
Positive rates of environmental and food allergen-specific IgE in dogs with AD and controls. The atopic group showed the highest positive frequencies for flea (B22), willow/cottonwood (t12/t14), *Cladosporium herbarum* (m3), sheep’s sorrel (w18), wool (e81), beef (f27), pumpkin (f225), milk (f2), pea (f14), and potato (f35). In contrast, the control group exhibited higher positive frequencies for flea (B22), wool (e81), willow/cottonwood (t12/t14), *Cladosporium herbarum* (m3), plantain (w9), beef (f27), pumpkin (f225), milk (f2), peanut (f13), and red deer (f867).

Regarding distribution, the most frequently positive allergens in the AD group were flea (B22), willow/cottonwood (t12/t14), *Cladosporium herbarum* (m3), sheep’s sorrel (w18), and wool (e81). For dietary allergens, beef (f27), pumpkin (f225), milk (f2), pea (f14), and potato (f35) were most often detected. The control group also showed reactivity to some of these allergens—particularly flea (B22), wool (e81), willow/cottonwood (t12/t14), and *C. herbarum* (m3)—but with overall lower frequencies. Detailed distributions are illustrated in Figure 2.

### 3.4. Cytokine Analysis

Serum concentrations of IL-4, IL-13, IL-31, and TGF-β1 were compared between groups. Mean values of IL-4, IL-13, and IL-31 were slightly higher in the AD group, whereas TGF-β1 was lower, but no statistically significant differences were observed for any cytokine (all *p* > 0.05). Detailed values (median [IQR]) are presented in Table 6.

## 4. Discussion

This study evaluated peripheral eosinophil counts, serum allergen-specific IgE, and selected cytokines in dogs with AD compared to clinically healthy controls. The results showed significant differences in allergen-specific IgE responses to storage mites and certain pollens, whereas peripheral eosinophil counts and serum cytokine levels did not differ significantly between groups. These findings emphasize the complexity of systemic immune alterations in canine AD and the challenges in identifying reliable blood-based biomarkers.

Eosinophils play a central role in allergic inflammation, particularly during late-phase hypersensitivity reactions. In humans, blood eosinophilia is often observed in asthma and AD and may correlate with disease severity in some cases [18,19]. However, eosinophil counts are highly variable and influenced by environmental exposure, circadian rhythm, and concurrent disease [20]. Veterinary studies similarly suggest that eosinophilia is more consistently associated with parasitic or hypersensitivity conditions than with AD [8]. In this study, eosinophil counts in AD dogs were not significantly different from controls, and most values fell within reference ranges. These results support the notion that systemic eosinophilia is not a sensitive marker for canine AD, as eosinophil infiltration occurs mainly within lesional skin rather than peripheral blood. Thus, blood eosinophil counts may be more useful for excluding parasitic disease or identifying comorbid allergic conditions rather than diagnosing AD.

In contrast, allergen-specific IgE testing demonstrated clearer diagnostic utility. Using a commercial immunoblot assay validated for reproducibility [16], significant elevations in IgE levels were detected against *Dermatophagoides pteronyssinus*, *Acarus siro*, *Tyrophagus putrescentiae*, and several pollens, including alder/birch, hazel, sycamore, willow/cottonwood, oak, plantain, and sheep’s sorrel. These results are consistent with previous studies identifying mites and pollens as key sensitizers in canine AD [21,22,23], although the specific allergen spectrum varies with geography and assay platform [24]. Importantly, positivity analysis revealed that *Acarus siro*, *Tyrophagus putrescentiae*, oak, and sheep’s sorrel showed significantly higher detection rates in AD dogs, with odds ratio and relative risk analyses confirming an association with disease, albeit of modest strength. This suggests that while IgE positivity alone cannot establish a diagnosis, it can help identify clinically relevant allergens for immunotherapy planning [22,25]. Notably, some dietary allergens with high IgE levels, such as pumpkin and pea, did not differ significantly between groups, underscoring the need for cautious interpretation and confirmation through elimination and challenge diets [26,27].

To further validate these associations, multivariate logistic regression analysis was conducted adjusting for potential confounding factors (age, sex, breed, and neutering status). The results confirmed that sensitization to storage mites, particularly *Acarus siro* (d70), remained independently associated with AD, whereas *Tyrophagus putrescentiae* (d72) showed a marginal trend, and pollen allergens lost statistical significance after adjustment. These findings suggest that storage mite exposure is a robust and independent risk factor for canine AD in this population, supporting its clinical relevance in diagnostic testing and allergen-specific immunotherapy.

The distribution of positivity further illustrates exposure dynamics. Both AD and control dogs showed frequent positivity to allergens such as flea, wool, willow/cottonwood, and *Cladosporium herbarum*, but rates were consistently higher in AD dogs. This pattern suggests background sensitization in non-atopic dogs, reflecting ubiquitous environmental exposure [23]. These findings reinforce that IgE testing should always be interpreted in the context of clinical history, environmental assessment, and therapeutic response rather than as a standalone diagnostic tool.

Cytokine analysis targeted IL-4, IL-13, IL-31, and TGF-β1, which play key roles in Th2-skewed inflammation and immune regulation [2,11,12,14,15]. In human AD, IL-4 and IL-13 promote IgE synthesis and barrier dysfunction, IL-31 induces pruritus via neuronal activation, and TGF-β1 mediates regulatory control [11,12,13,14]. In this study, dogs with AD showed nonsignificant trends toward higher IL-4, IL-13, and IL-31, and lower TGF-β1. These trends partially align with previous reports of increased IL-13 and IL-31 and decreased TGF-β1 in canine AD [10], though results are inconsistent across studies [14,28]. The lack of statistical significance may reflect the localized activity of cytokines in lesional skin, heterogeneity in disease stage, or limited sample size [28]. Indeed, prior work indicates that skin biopsy cytokine profiles more accurately reflect disease mechanisms than serum levels [14].

Despite the absence of significant differences, the observed trends have clinical relevance. IL-31 is a well-established therapeutic target, with oclacitinib and lokivetmab effectively reducing pruritus by modulating IL-31-mediated pathways [29,30]. Similarly, the downward trend in TGF-β1 may suggest impaired immune regulation contributing to disease chronicity, highlighting an avenue for further investigation. These findings illustrate that systemic cytokine assessment, while limited diagnostically, may still provide translational insights into therapeutic mechanisms.

Taken together, the results indicate that no single systemic biomarker reliably diagnoses canine AD. Eosinophil counts are nondiscriminatory, cytokine differences in serum are subtle, and IgE positivity is informative but not definitive. However, combined assessment provides complementary information. Among these, serum allergen-specific IgE testing appears most clinically useful in guiding allergen selection for immunotherapy, particularly in Korean dogs where storage mites and pollens emerged as dominant sensitizers. The findings also emphasize the importance of conducting regional allergen profiling, as sensitization patterns differ across climates and environments [14,23].

The study has several limitations. The possibility of concurrent food allergy could not be fully excluded, although Favrot’s criteria were applied to confirm AD diagnosis. The sample size was moderate and derived from a single region, which may limit generalizability. Additionally, potential sources of bias should be considered, including selection bias due to hospital-based recruitment and possible information bias arising from owner-reported histories. Nevertheless, all dogs underwent standardized diagnostic evaluation based on Favrot’s criteria, minimizing misclassification risk. Cytokine analysis was restricted to four targets; inclusion of additional cytokines such as IL-5, IFN-γ, or IL-10 could provide a broader view of immune dysregulation. Moreover, only serum samples were evaluated, whereas tissue-based cytokine profiling might better capture local immunological changes. To address these limitations, future studies should involve multicenter cohorts with larger sample sizes, stratification by disease stage, and integration of both systemic and local immune markers. Comparative analyses across regions will be essential to refine allergen panels for diagnostic and therapeutic use, and longitudinal monitoring of cytokines and allergen-specific responses could further clarify their value in disease progression and treatment response.

## 5. Conclusions

This study demonstrated that peripheral eosinophil counts and serum cytokines have limited discriminatory value for canine AD, whereas allergen-specific IgE testing showed significant associations with storage mites and selected pollens. These results highlight the relevance of region-specific allergen profiling in guiding immunotherapy and indicate that systemic cytokine measurements may not reliably reflect local immune dynamics. Collectively, the findings provide a foundation for future work aimed at refining diagnostic strategies and developing targeted therapies to improve the clinical management of canine AD.

## Figures and Tables

**Table 1:** Classification criteria of alternate scoring method.

ASM Class	IgE Levels (AU/mL)	Evaluation	Clinical Interpretation
0	<0.35	Negative	Allergic reaction excluded
1	≥0.35–<0.7	Weak positive	Possible risk of allergic reaction
2	≥0.7–<3.5		
3	≥3.5–<17.5	Strong positive	Clinically relevant level
4	≥17.5–<50		
5	≥50–<100	Very strong positive	Highly clinically relevant level
6	≥100		

All IgE values ≥ 0.35 AU/mL were considered positive. ASM; alternate scoring method.

**Table 2:** Characteristics of the dogs with atopic dermatitis and controls in this study.

Characteristics	AD Group				Control Group			
	N	%	Mean ± SD		N	%	Mean ± SD	
Age (overall)	65	100	6.34 ± 3.99		28	100	3.59 ± 4.12	
<1 year	3	5			12	43		
1–6 year	34	52			10	36		
>6 years	28	43			6	21		
Sex								
Male	6	9			8	29		
Castrated male	33	51			10	36		
Female	8	12			4	14		
Spayed female	18	28			6	21		
Breeds								
	Bichon Frise (3), Boston Terrier (4), Chihuahua (1), Chow Chow (1), Cocker Spaniel (1), French Bulldog (1), Japanese Spitz (1), Labrador Retriever (2), Maltese (20), Mixed (5), Pomeranian (4), Poodle (7), Shih Tzu (8), Spitz (1), Welsh Corgi (1), Yorkshire Terrier (5)				Bichon Frise (3), Chihuahua (1), Golden Retriever (3), Maltese (6), Miniature Pinscher (1), Mixed (2), Pomeranian (5), Poodle (5), Yorkshire Terrier (2)			

AD; atopic dermatitis, SD; standard deviation.

**Table 3:** Comparison of allergen-specific IgE levels in dogs with atopic dermatitis and healthy controls.

Classification		Code	Allergens	AD Group Median [IQR]	Control Group Median [IQR]	*p*
Environmental allergens	Mite	d1	*Dermatophagoides pteronyssinus*	0.15 [0.15–0.17]	0.15 [0.15–0.15]	0.023 *
		d2	*Dermatophagoides farinae*	0.15 [0.15–0.15]	0.15 [0.15–0.15]	0.051
		d70	*Acarus siro*	0.15 [0.15–0.15]	0.15 [0.15–0.15]	0.038 *
		d72	*Tyrophagus putrescentiae*	0.15 [0.15–0.15]	0.15 [0.15–0.15]	0.016 *
	Pollen	t2/t3	Alder/Birch	0.15 [0.15–0.20]	0.15 [0.15–0.15]	0.003 **
		t4	Hazel	0.15 [0.15–0.15]	0.15 [0.15–0.15]	0.016 *
		t11	Maple leaf sycamore	0.26 [0.15–0.34]	0.15 [0.15–0.21]	0.003 **
		t12/t14	Willow/Cottonwood	0.34 [0.15–0.44]	0.15 [0.15–0.27]	0.014 *
		t7	Oak	0.15 [0.15–0.20]	0.15 [0.15–0.15]	0.005 **
		t16	White pine	0.15 [0.15–0.15]	0.15 [0.15–0.15]	0.108
		t19	Acacia	0.15 [0.15–0.15]	0.15 [0.15–0.15]	0.585
		t15	White ash	0.15 [0.15–0.15]	0.15 [0.15–0.15]	0.793
		w1	Common ragweed	0.15 [0.15–0.15]	0.15 [0.15–0.15]	0.718
		w9	Plantain	0.20 [0.15–0.34]	0.15 [0.15–0.20]	0.005 **
		w6	Mugwort	0.15 [0.15–0.15]	0.15 [0.15–0.15]	0.238
		w18	Sheep’s sorrel	0.15 [0.15–0.51]	0.15 [0.15–0.15]	0.007 **
		g2	Bermuda grass	0.15 [0.15–0.15]	0.15 [0.15–0.15]	0.065
		g3/g6	Orchard grass/ Timothy grass	0.15 [0.15–0.15]	0.15 [0.15–0.15]	0.443
		g5	Ryegrass	0.15 [0.15–0.15]	0.15 [0.15–0.15]	0.281
		g12	Cultivated rye	0.15 [0.15–0.15]	0.15 [0.15–0.15]	0.546
	Molds	m1	*Penicillium notatum*	0.15 [0.15–0.15]	0.15 [0.15–0.15]	0.512
		m3	*Cladosporium herbarum*	0.19 [0.15–0.38]	0.15 [0.15–0.29]	0.031 *
		m2	*Aspergillus fumigatus*	0.15 [0.15–0.34]	0.15 [0.15–0.15]	0.111
		m6	*Candida albicans*	0.15 [0.15–0.15]	0.15 [0.15–0.15]	0.098
		m5	*Alternaria alternata*	0.15 [0.15–0.15]	0.15 [0.15–0.15]	0.455
		m227	*Malassezia pachydermatis*	0.15 [0.15–0.15]	0.15 [0.15–0.15]	0.922
	Insect	B22	Flea	0.15 [0.15–0.93]	0.15 [0.15–0.21]	0.187
		i6	Cockroach	0.15 [0.15–0.15]	0.15 [0.15–0.15]	0.443
	Others (Epi/indoor)	e1/e2	Cat epithelium/ Cat dander	0.18 [0.15–0.34]	0.15 [0.15–0.15]	0.001 **
		e81	Wool	0.15 [0.15–0.43]	0.15 [0.15–0.15]	0.242
		ex1	Feather mix	0.15 [0.15–0.15]	0.15 [0.15–0.15]	1.000
Food allergens	Meats	f26	Pork	0.15 [0.15–0.15]	0.15 [0.15–0.15]	0.914
		f27	Beef	0.19 [0.15–1.90]	0.16 [0.15–1.32]	0.817
		f581	Duck meat	0.15 [0.15–0.15]	0.15 [0.15–0.15]	0.512
		f83	Chicken	0.15 [0.15–0.15]	0.15 [0.15–0.15]	0.906
		f88	Lamb meat	0.15 [0.15–0.15]	0.15 [0.15–0.15]	0.881
		f284	Turkey meat	0.15 [0.15–0.15]	0.15 [0.15–0.15]	1.000
		f867	Red deer	0.15 [0.15–0.15]	0.15 [0.15–0.15]	0.572
	Grains, Beans	f4	Wheat	0.15 [0.15–0.15]	0.15 [0.15–0.15]	0.182
		f8	Corn	0.15 [0.15–0.15]	0.15 [0.15–0.15]	0.415
		f9	Rice	0.15 [0.15–0.15]	0.15 [0.15–0.15]	0.906
		f12	Pea	0.16 [0.15–0.25]	0.15 [0.15–0.16]	0.017 *
		f14	Soy bean	0.15 [0.15–0.24]	0.15 [0.15–0.15]	0.026 *
	Fruit, Vegetable	f31	Carrot	0.15 [0.15–0.15]	0.15 [0.15–0.15]	0.901
		f35	Potato	0.15 [0.15–0.20]	0.15 [0.15–0.16]	0.141
		f54	Sweet potato	0.15 [0.15–0.15]	0.15 [0.15–0.15]	0.250
		f225	Pumpkin	0.27 [0.15–0.52]	0.15 [0.15–0.27]	0.021 *
		f25	Tomato	0.15 [0.15–0.15]	0.15 [0.15–0.15]	0.095
		f49	Apple	0.15 [0.15–0.15]	0.15 [0.15–0.15]	0.016 *
	Yeast	f45	Yeast, baker’s	0.15 [0.15–0.15]	0.15 [0.15–0.15]	0.537
	Dairy products	f1	Egg white	0.15 [0.15–0.15]	0.15 [0.15–0.15]	0.637
		f75	Egg yolk	0.16 [0.15–0.28]	0.16 [0.15–0.28]	0.329
		f2	Milk	0.15 [0.15–0.87]	0.15 [0.15–0.15]	0.261
		f81	Cheddar cheese	0.15 [0.15–0.15]	0.15 [0.15–0.15]	0.372
	Seafood	f23/f24	Crab/shrimp	0.15 [0.15–0.15]	0.15 [0.15–0.15]	0.793
		f3	Codfish	0.15 [0.15–0.15]	0.15 [0.15–0.15]	0.214
		f40	Tuna	0.15 [0.15–0.15]	0.15 [0.15–0.15]	0.549
		f41	Salmon	0.15 [0.15–0.15]	0.15 [0.15–0.15]	0.549
		f206	Mackerel	0.15 [0.15–0.15]	0.15 [0.15–0.15]	1.000
	Nut	f13	Peanut	0.15 [0.15–0.27]	0.15 [0.15–0.27]	0.454

Values are expressed as median [interquartile range (IQR)]. AD: atopic dermatitis. Asterisks indicate significant differences (* *p* < 0.05; ** *p* < 0.01).

**Table 4:** Odds ratio and relative risk of significant difference positive rate allergens.

Environmental	Code	Allergens	Odds Ratio	95% CI		Relative Risk	95% CI	
				Lower Bound	Upper Bound		Lower Bound	Upper Bound
Mites	d70	*Acarus siro*	7.412	0.924	59.428	1.427	1.156	1.762
	d72	*Tyrophagus putrescentiae*	8.100	1.014	64.685	1.444	1.174	1.776
Pollens	t7	Oak				1.519	1.299	1.775
	w18	Sheep’s sorrel	4.979	1.070	23.164	1.398	1.117	1.750

CI: confidence interval. Odds ratios could not be calculated for oak (t7) due to the absence of positive cases in the control group.

**Table 5:** Adjusted odds ratios for associations between allergen positivity and AD in dogs based on multivariate logistic regression analysis.

Environmental	Code	Allergens	Odds Ratio	95% CI		*p*
				Lower Bound	Upper Bound	
Mites	d70	*Acarus siro*	3.45	1.08	10.98	0.037 *
	d72	*Tyrophagus putrescentiae*	2.86	0.95	8.55	0.061
Pollens	t7	Oak	1.21	0.42	3.45	0.718
	w18	Sheep’s sorrel	0.91	0.33	2.52	0.856

CI: confidence interval. Results are based on multivariate logistic regression analysis adjusted for age, sex, breed, and neutering status. Asterisks indicate significant differences (* *p* < 0.05).

**Table 6:** Comparisons of cytokine expression in dogs with atopic dermatitis and controls in this study.

Cytokines	AD Group (Median [IQR])	Control Group (Median [IQR])	*p*
IL-4 (pg/mL)	215.20 [156.51–341.72]	212.94 [146.48–250.38]	0.436
IL-13 (pg/mL)	139.05 [91.43–267.98]	122.61 [91.84–165.84]	0.247
IL-31(pg/mL)	109.70 [70.79–223.02]	103.32 [82.52–137.05]	0.960
TGF-β1(pg/mL)	406.28 [283.75–598.93]	519.68 [334.39–628.41]	0.128

Values are expressed as median [interquartile range (IQR)]. IL-4 and IL-31 were analyzed using the independent *t*-test, whereas IL-13 and TGF-β1 were analyzed using the Mann–Whitney U test. AD; atopic dermatitis, *p*; statistical significance, IL; interleukin, TGF-β1; transforming growth factor β1.

## Data Availability

The datasets generated and analyzed during the current study are available from the corresponding author upon reasonable request.

## Supplementary Materials

The following supporting information can be downloaded at: https://www.mdpi.com/article/10.3390/ani15213219/s1, Table S1: Classification of tested environmental and food allergens in this study; Table S2: Comparison of environmental and food allergen-specific IgE positive rates (≥ Class 2) in dogs with atopic dermatitis and controls in this study.
