# Supplementary material for: Evaluation of Total Eosinophil Counts, Serum Allergen-Specific IgE and Related Cytokines in Dogs with Atopic Dermatitis
Source: Animals (Basel). 2025 Nov 5;15(21):3219. doi: 10.3390/ani15213219 (PMC12609046; doi:10.3390/ani15213219)
Supplement: Supplementary file 1 [file animals-15-03219-s001.zip › animals-3957399-supplementary.pdf]

Supplementary Table S1. Classification of tested environmental and food allergens in this study.

Classification		Code	Allergens
Environmental allergens	**Mite**	d1	*Dermatophagoides pteronyssinus*
		d2	*Dermatophagoides farinae*
		d70	*Acarus siro*
		d72	*Tyrophagus putrescentiae*
	**Pollen**	t2/t3	Alder/Birch
		t4	Hazel
		t11	Maple leaf sycamore
		t12/t14	Willow/Cottonwood
		t7	Oak
		t16	White Pine
		t19	Acacia
		t15	White ash
		w1	Common Ragweed
		w9	Plantain
		w6	Mugwort
		w18	Sheep's sorrel
		g2	Bermuda grass
		g3/g6	Orchard grass/Timothy grass
		g5	Ryegrass
		g12	Cultivated rye
	**Molds**	m1	*Penicillium notatum*
		m3	*Cladosporium herbarum*
		m2	*Aspergillus fumigatus*
		m6	*Candida albicans*
		m5	*Alternaria alternata*
		m227	*Malassezia pachydermatis*
	**Insect**	B22	Flea
		i6	Cockroach
	**Others (Epi/indoor)**	e1/e2	Cat Epithelium/Cat Dander
		e81	Wool

		ex1	Feather mix
Food allergens	**Meats**	f26	Pork
		f27	Beef
		f581	Duck meat
		f83	Chicken
		f88	Lamb meat
		f284	Turkey meat
		f867	Red deer
	**Grains, Beans**	f4	Wheat
		f8	Corn
		f9	Rice
		f12	Pea
		f14	Soy bean
	**Fruit, Vegetable**	f31	Carrot
		f35	Potato
		f54	Sweet potato
		f225	Pumpkin
		f25	Tomato
		f49	Apple
	**Yeast**	f45	Yeast, baker's
	**Dairy products**	f1	Egg white
		f75	Egg yolk
		f2	Milk
		f81	Cheddar cheese
	**Seafood**	f23/f24	Crab/shrimp
		f3	Codfish
		f40	Tuna
		f41	Salmon
		f206	Mackerel
	**Nut**	f13	Peanut

Supplementary Table S2. Comparison of environmental and food allergen-specific IgE positive rates (≥ Class 2) in dogs with atopic dermatitis and con trols in this study.

Classification		Code	Allergens	AD Group (%)	Control Group (%)	P	Odds ratio (95% CI)
**Environmental allergens**	**Mite**	d1	*Dermatophagoides pteronyssinus*	18.5	7.1	0.215	2.94 (0.61–14.13)
		d2	*Dermatophagoides farinae*	23.1	7.1	0.084	3.90 (0.83–18.37)
		d70	*Acarus siro*	21.5	3.6	0.033*	7.41 (0.92–59.43)
		d72	*Tyrophagus putrescentiae*	23.1	3.6	0.033*	8.10 (1.01–64.69)
	**Pollen**	t2/t3	Alder/Birch	12.3	0.0	0.1	8.43 (0.47–151.21)
		t4	Hazel	12.3	0.0	0.1	8.43 (0.47–151.21)
		t11	Maple leaf sycamore	24.6	7.1	0.083	4.24 (0.91–19.90)
		t12/t14	Willow/Cottonwood	32.3	14.3	0.081	2.86 (0.88–9.31)
		t7	Oak	16.9	0.0	0.03*	12.03 (0.68–211.60)
		t16	White Pine	7.7	3.6	0.664	2.25 (0.25–20.20)
		t19	Acacia	6.2	3.6	1	1.77 (0.19–16.59)
		t15	White ash	4.6	3.6	1	1.31 (0.13–13.13)
		w1	Common Ragweed	7.7	7.1	1	1.08 (0.20–5.95)
		w9	Plantain	20.0	10.7	0.375	2.08 (0.54–7.98)
		w6	Mugwort	9.2	0.0	0.173	6.23 (0.34–114.41)
		w18	Sheep's sorrel	27.7	7.1	0.029*	4.98 (1.07–23.16)
		g2	Bermuda grass	6.2	3.6	1	1.77 (0.19–16.59)
		g3/g6	Orchard grass/Timothy grass	6.2	3.6	1	1.77 (0.19–16.59)
		g5	Ryegrass	6.2	3.6	1	1.77 (0.19–16.59)
		g12	Cultivated rye	7.7	3.6	0.664	2.25 (0.25–20.20)
	**Molds**	m1	*Penicillium notatum*	1.5	0.0	1	1.33 (0.05–33.54)
		m3	*Cladosporium herbarum*	30.8	14.3	0.124	2.67 (0.82–8.70)
		m2	*Aspergillus fumigatus*	12.3	3.6	0.269	3.79 (0.45–31.85)
		m6	*Candida albicans*	9.2	0.0	0.173	6.23 (0.34–114.41)
		m5	*Alternaria alternata*	7.7	3.6	0.664	2.25 (0.25–20.20)
		m227	*Malassezia pachydermatis*	9.2	10.7	1	0.85 (0.20–3.66)
	**Insect**	B22	Flea	35.4	25.0	0.326	1.64 (0.61–4.44)
		i6	Cockroach	6.2	3.6	1	1.77 (0.19–16.59)

	**Others (Epi/indoor)**	e1/e2	Cat Epithelium/Cat Dander	20.0	3.6	0.057	6.75 (0.84–54.38)
		e81	Wool	27.7	17.9	0.434	1.76 (0.58–5.34)
		ex1	Feather mix	0.0	0.0	–	–
**Food allergens**	**Meats**	**f26**	**Pork**	10.8	14.3	0.729	0.72 (0.19–2.70)
		f27	Beef	43.1	35.7	0.646	1.36 (0.55–3.40)
		f581	Duck meat	0.0	0.0	–	–
		f83	Chicken	4.6	0.0	0.551	3.19 (0.16–63.87)
		f88	Lamb meat	9.2	10.7	1	0.85 (0.20–3.66)
		f284	Turkey meat	0.0	0.0	–	–
		f867	Red deer	18.5	17.9	0.945	1.04 (0.33–3.30)
	**Grains, Beans**	f4	Wheat	4.6	0.0	0.551	3.19 (0.16–63.87)
		f8	Corn	7.7	3.6	0.664	2.25 (0.25–20.20)
		f9	Rice	6.2	3.6	1	1.77 (0.19–16.59)
		f12	Pea	13.8	0.0	0.053	9.58 (0.54–170.61)
		f14	Soy bean	23.1	7.1	0.084	3.90 (0.83–18.37)
	**Fruit, Vegetable**	f31	Carrot	18.5	17.9	1	1.04 (0.33–3.30)
		f35	Potato	23.1	14.3	0.41	1.80 (0.54–6.01)
		f54	Sweet potato	4.6	0.0	0.551	3.19 (0.16–63.87)
		f225	Pumpkin	40.0	21.4	0.084	2.44 (0.87–6.85)
		f25	Tomato	10.8	3.6	0.428	3.26 (0.38–27.82)
		f49	Apple	7.7	0.0	0.318	5.18 (0.28–96.96)
	**Yeast**	f45	Yeast, baker's	0.0	0.0	–	–
	**Dairy products**	f1	Egg white	3.1	7.1	0.581	0.41 (0.06–3.09)
		f75	Egg yolk	23.1	14.3	0.41	1.80 (0.54–6.01)
		f2	Milk	35.4	21.4	0.183	2.01 (0.71–5.66)
		f81	Cheddar cheese	1.5	3.6	0.514	0.42 (0.03–6.99)
	**Seafood**	f23/f24	Crab/shrimp	12.3	7.1	0.718	1.82 (0.36–9.20)
		f3	Codfish	12.3	3.6	0.269	3.79 (0.45–31.85)
		f40	Tuna	1.5	3.6	0.514	0.42 (0.03–6.99)
		f41	Salmon	1.5	3.6	0.514	0.42 (0.03–6.99)
		f206	Mackerel	0.0	0.0	–	–
	**Nut**	f13	Peanut	16.9	21.4	0.606	0.75 (0.25–2.27)

AD, atopic dermatitis; P, statistical significance; Positive rate, percentage of animals classified as ASM Class 2 or higher for each allergen; * indicates significant difference at $P < 0.05$; – indicates allergen not detected in either group. Odds ratios were calculated with 95% confidence intervals; when zero counts occurred in one group, the Haldane–Anscombe correction (0.5 continuity correction) was applied, whereas allergens with zero counts in both groups were not estimable.
